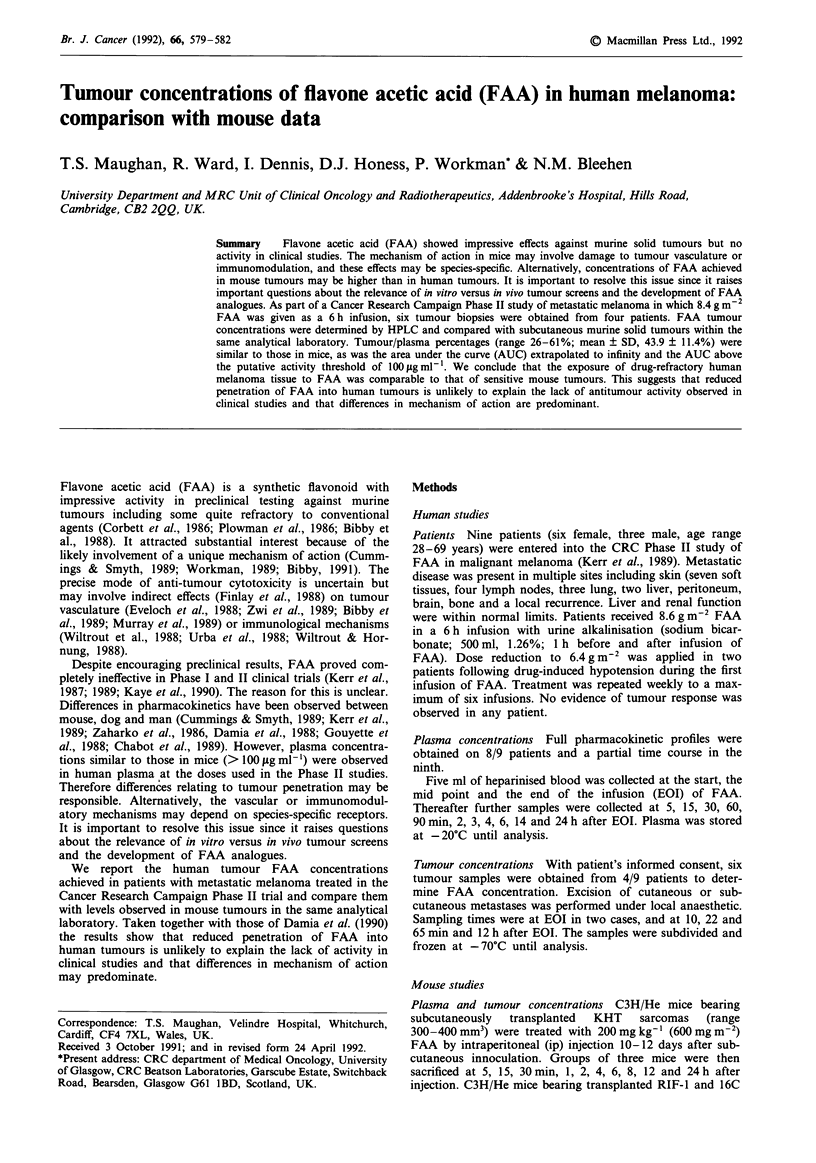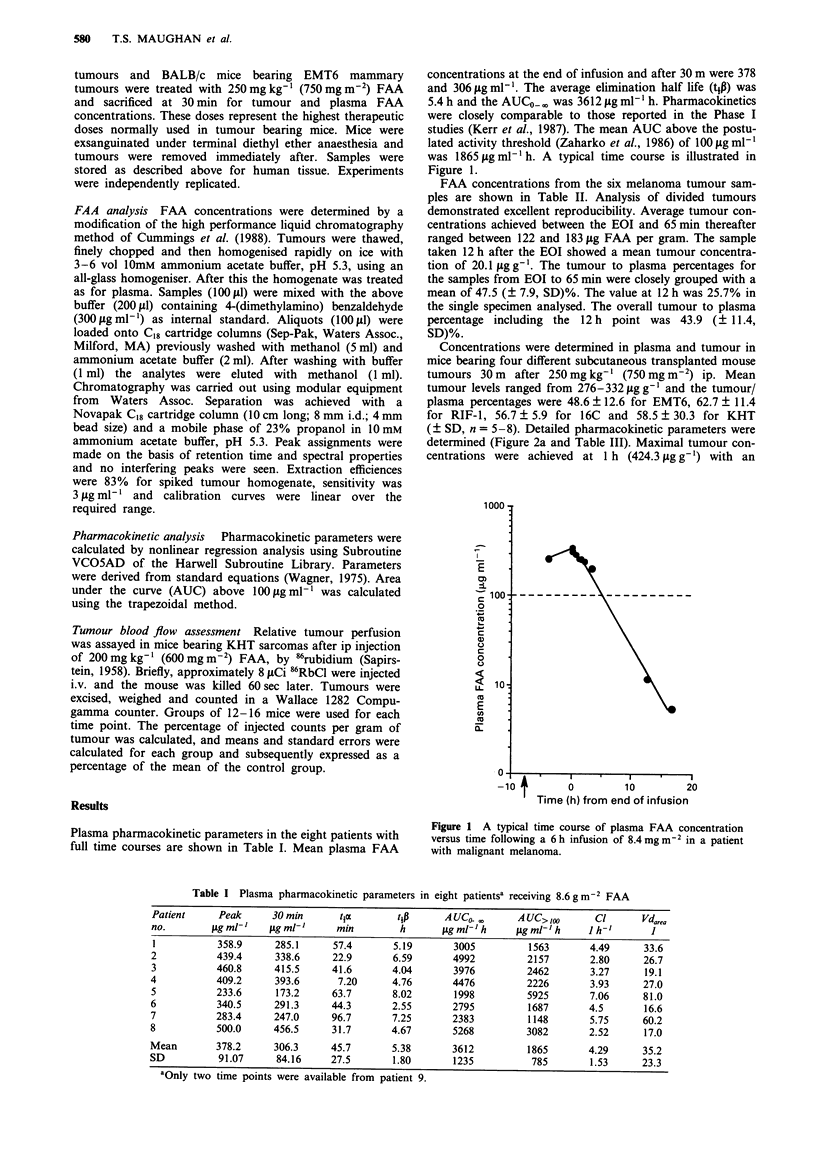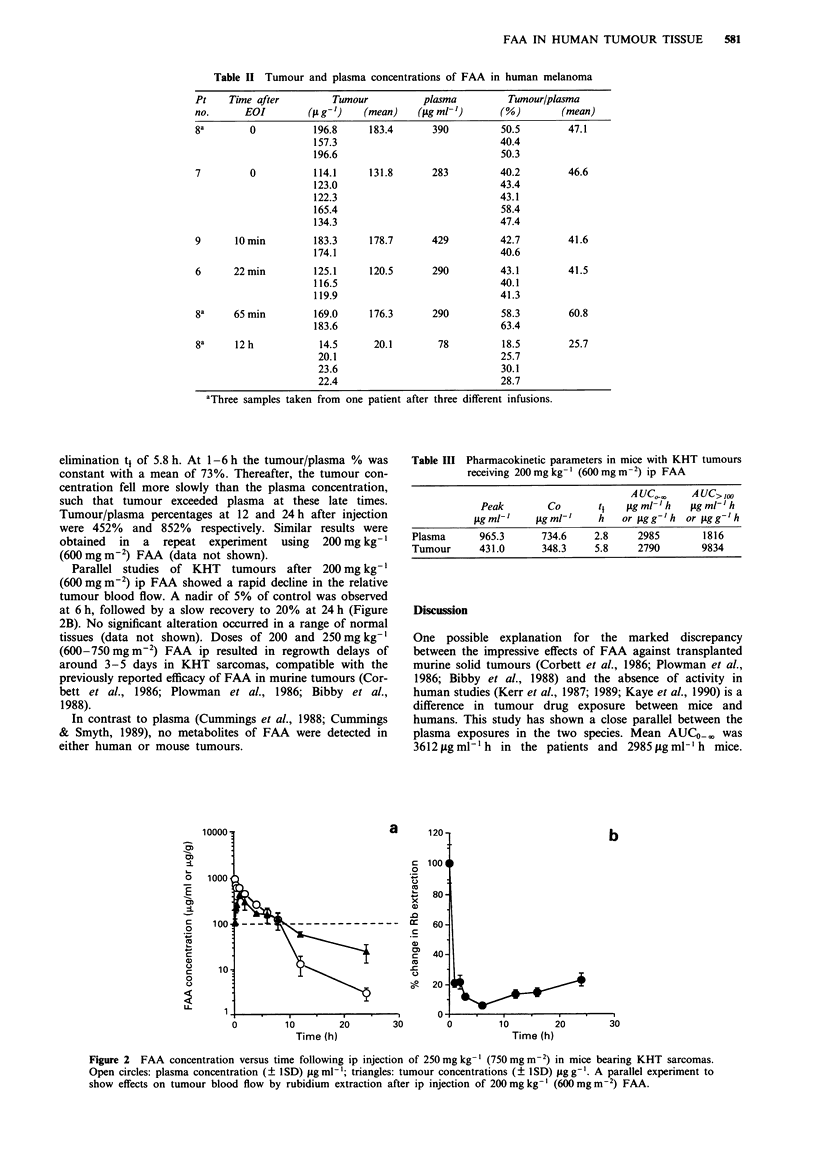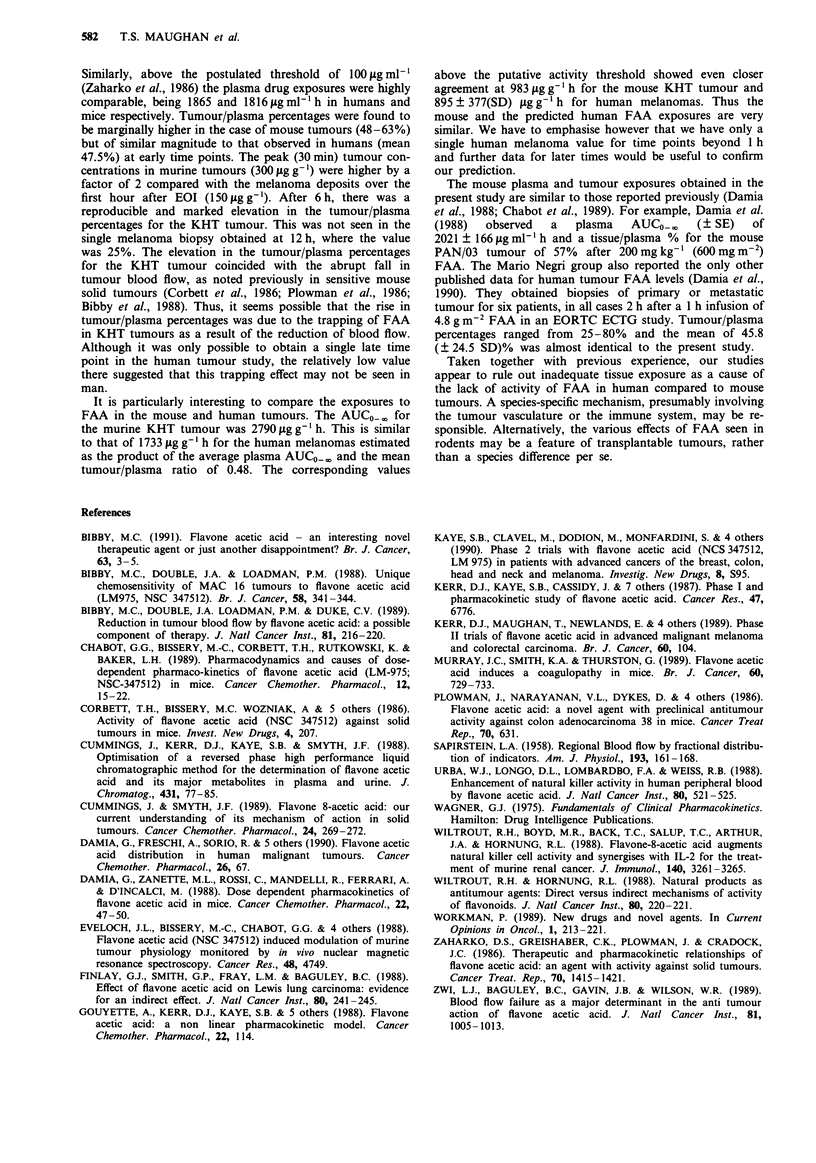# Tumour concentrations of flavone acetic acid (FAA) in human melanoma: comparison with mouse data.

**DOI:** 10.1038/bjc.1992.317

**Published:** 1992-09

**Authors:** T. S. Maughan, R. Ward, I. Dennis, D. J. Honess, P. Workman, N. M. Bleehen

**Affiliations:** University Department, Addenbrooke's Hospital, Cambridge, UK.

## Abstract

Flavone acetic acid (FAA) showed impressive effects against murine solid tumours but no activity in clinical studies. The mechanism of action in mice may involve damage to tumour vasculature or immunomodulation, and these effects may be species-specific. Alternatively, concentrations of FAA achieved in mouse tumours may be higher than in human tumours. It is important to resolve this issue since it raises important questions about the relevance of in vitro versus in vivo tumour screens and the development of FAA analogues. As part of a Cancer Research Campaign Phase II study of metastatic melanoma in which 8.4 g m-2 FAA was given as a 6 h infusion, six tumour biopsies were obtained from four patients. FAA tumour concentrations were determined by HPLC and compared with subcutaneous murine solid tumours within the same analytical laboratory. Tumour/plasma percentages (range 26-61%; mean +/- SD, 43.9 +/- 11.4%) were similar to those in mice, as was the area under the curve (AUC) extrapolated to infinity and the AUC above the putative activity threshold of 100 micrograms ml-1. We conclude that the exposure of drug-refractory human melanoma tissue to FAA was comparable to that of sensitive mouse tumours. This suggests that reduced penetration of FAA into human tumours is unlikely to explain the lack of antitumour activity observed in clinical studies and that differences in mechanism of action are predominant.


Br. J. Cancer (1992), 66, 579-582 © Macmillan Press Ltd., 1992

### Tumour concentrations of flavone acetic acid (FAA) in human melanoma: comparison with mouse data

T.S. Maughan, R. Ward, I. Dennis, D.J. Honess, P. Workman* & N.M. Bleehen

*University Department and MRC Unit of Clinical Oncology and Radiotherapeutics, Addenbrooke's Hospital, Hills Road, Cambridge, CB2 2QQ, UK.*

**Summary** Flavone acetic acid (FAA) showed impressive effects against murine solid tumours but no activity in clinical studies. The mechanism of action in mice may involve damage to tumour vasculature or immunomodulation, and these effects may be species-specific. Alternatively, concentrations of FAA achieved in mouse tumours may be higher than in human tumours. It is important to resolve this issue since it raises important questions about the relevance of *in vitro* versus *in vivo* tumour screens and the development of FAA analogues. As part of a Cancer Research Campaign Phase II study of metastatic melanoma in which 8.4 g m$^{-2}$ FAA was given as a 6 h infusion, six tumour biopsies were obtained from four patients. FAA tumour concentrations were determined by HPLC and compared with subcutaneous murine solid tumours within the same analytical laboratory. Tumour/plasma percentages (range 26-61%; mean ± SD, 43.9 ± 11.4%) were similar to those in mice, as was the area under the curve (AUC) extrapolated to infinity and the AUC above the putative activity threshold of 100 μg ml$^{-1}$. We conclude that the exposure of drug-refractory human melanoma tissue to FAA was comparable to that of sensitive mouse tumours. This suggests that reduced penetration of FAA into human tumours is unlikely to explain the lack of antitumour activity observed in clinical studies and that differences in mechanism of action are predominant.

Flavone acetic acid (FAA) is a synthetic flavonoid with impressive activity in preclinical testing against murine tumours including some quite refractory to conventional agents (Corbett *et al.*, 1986; Plowman *et al.*, 1986; Bibby et al., 1988). It attracted substantial interest because of the likely involvement of a unique mechanism of action (Cummings & Smyth, 1989; Workman, 1989; Bibby, 1991). The precise mode of anti-tumour cytotoxicity is uncertain but may involve indirect effects (Finlay *et al.*, 1988) on tumour vasculature (Eveloch *et al.*, 1988; Zwi *et al.*, 1989; Bibby *et al.*, 1989; Murray *et al.*, 1989) or immunological mechanisms (Wiltrout et al., 1988; Urba *et al.*, 1988; Wiltrout & Hornung, 1988).

Despite encouraging preclinical results, FAA proved completely ineffective in Phase I and II clinical trials (Kerr *et al.*, 1987; 1989; Kaye *et al.*, 1990). The reason for this is unclear. Differences in pharmacokinetics have been observed between mouse, dog and man (Cummings & Smyth, 1989; Kerr *et al.*, 1989; Zaharko *et al.*, 1986, Damia *et al.*, 1988; Gouyette *et al.*, 1988; Chabot *et al.*, 1989). However, plasma concentrations similar to those in mice (> 100 μg ml$^{-1}$) were observed in human plasma at the doses used in the Phase II studies. Therefore differences relating to tumour penetration may be responsible. Alternatively, the vascular or immunomodulatory mechanisms may depend on species-specific receptors. It is important to resolve this issue since it raises questions about the relevance of *in vitro* versus *in vivo* tumour screens and the development of FAA analogues.

We report the human tumour FAA concentrations achieved in patients with metastatic melanoma treated in the Cancer Research Campaign Phase II trial and compare them with levels observed in mouse tumours in the same analytical laboratory. Taken together with those of Damia *et al.* (1990) the results show that reduced penetration of FAA into human tumours is unlikely to explain the lack of activity in clinical studies and that differences in mechanism of action may predominate.

Correspondence: T.S. Maughan, Velindre Hospital, Whitchurch, Cardiff, CF4 7XL, Wales, UK.
Received 3 October 1991; and in revised form 24 April 1992.
*Present address: CRC department of Medical Oncology, University of Glasgow, CRC Beatson Laboratories, Garscube Estate, Switchback Road, Bearsden, Glasgow G61 1BD, Scotland, UK.

### Methods

### *Human studies*

*Patients* Nine patients (six female, three male, age range 28-69 years) were entered into the CRC Phase II study of FAA in malignant melanoma (Kerr *et al.*, 1989). Metastatic disease was present in multiple sites including skin (seven soft tissues, four lymph nodes, three lung, two liver, peritoneum, brain, bone and a local recurrence. Liver and renal function were within normal limits. Patients received 8.6 g m$^{-2}$ FAA in a 6 h infusion with urine alkalinisation (sodium bicarbonate; 500 ml, 1.26%; 1 h before and after infusion of FAA). Dose reduction to 6.4 g m$^{-2}$ was applied in two patients following drug-induced hypotension during the first infusion of FAA. Treatment was repeated weekly to a maximum of six infusions. No evidence of tumour response was observed in any patient.

*Plasma concentrations* Full pharmacokinetic profiles were obtained on 8/9 patients and a partial time course in the ninth.

Five ml of heparinised blood was collected at the start, the mid point and the end of the infusion (EOI) of FAA. Thereafter further samples were collected at 5, 15, 30, 60, 90 min, 2, 3, 4, 6, 14 and 24 h after EOI. Plasma was stored at −20°C until analysis.

*Tumour concentrations* With patient's informed consent, six tumour samples were obtained from 4/9 patients to determine FAA concentration. Excision of cutaneous or subcutaneous metastases was performed under local anaesthetic. Sampling times were at EOI in two cases, and at 10, 22 and 65 min and 12 h after EOI. The samples were subdivided and frozen at −70°C until analysis.

### *Mouse studies*

*Plasma and tumour concentrations* C3H/He mice bearing subcutaneously transplanted KHT sarcomas (range 300-400 mm$^3$) were treated with 200 mg kg$^{-1}$ (600 mg m$^{-2}$) FAA by intraperitoneal (ip) injection 10-12 days after subcutaneous innoculation. Groups of three mice were then sacrificed at 5, 15, 30 min, 1, 2, 4, 6, 8, 12 and 24 h after injection. C3H/He mice bearing transplanted RIF-1 and 16C

tumours and BALB/c mice bearing EMT6 mammary tumours were treated with 250 mg $kg^{-1}$ (750 mg $m^{-2}$) FAA and sacrificed at 30 min for tumour and plasma FAA concentrations. These doses represent the highest therapeutic doses normally used in tumour bearing mice. Mice were exsanguinated under terminal diethyl ether anaesthesia and tumours were removed immediately after. Samples were stored as described above for human tissue. Experiments were independently replicated.

*FAA analysis* FAA concentrations were determined by a modification of the high performance liquid chromatography method of Cummings *et al.* (1988). Tumours were thawed, finely chopped and then homogenised rapidly on ice with 3–6 vol 10mM ammonium acetate buffer, pH 5.3, using an all-glass homogeniser. After this the homogenate was treated as for plasma. Samples (100 μl) were mixed with the above buffer (200 μl) containing 4-(dimethylamino) benzaldehyde (300 μg $ml^{-1}$) as internal standard. Aliquots (100 μl) were loaded onto $C_{18}$ cartridge columns (Sep-Pak, Waters Assoc., Milford, MA) previously washed with methanol (5 ml) and ammonium acetate buffer (2 ml). After washing with buffer (1 ml) the analytes were eluted with methanol (1 ml). Chromatography was carried out using modular equipment from Waters Assoc. Separation was achieved with a Novapak $C_{18}$ cartridge column (10 cm long; 8 mm i.d.; 4 mm bead size) and a mobile phase of 23% propanol in 10 mM ammonium acetate buffer, pH 5.3. Peak assignments were made on the basis of retention time and spectral properties and no interfering peaks were seen. Extraction efficiences were 83% for spiked tumour homogenate, sensitivity was 3 μg $ml^{-1}$ and calibration curves were linear over the required range.

*Pharmacokinetic analysis* Pharmacokinetic parameters were calculated by nonlinear regression analysis using Subroutine VCO5AD of the Harwell Subroutine Library. Parameters were derived from standard equations (Wagner, 1975). Area under the curve (AUC) above 100 μg $ml^{-1}$ was calculated using the trapezoidal method.

*Tumour blood flow assessment* Relative tumour perfusion was assayed in mice bearing KHT sarcomas after ip injection of 200 mg $kg^{-1}$ (600 mg $m^{-2}$) FAA, by $^{86}$rubidium (Sapirstein, 1958). Briefly, approximately 8 μCi $^{86}$RbCl were injected i.v. and the mouse was killed 60 sec later. Tumours were excised, weighed and counted in a Wallace 1282 Compugamma counter. Groups of 12–16 mice were used for each time point. The percentage of injected counts per gram of tumour was calculated, and means and standard errors were calculated for each group and subsequently expressed as a percentage of the mean of the control group.

### Results

Plasma pharmacokinetic parameters in the eight patients with full time courses are shown in Table I. Mean plasma FAA concentrations at the end of infusion and after 30 m were 378 and 306 μg $ml^{-1}$. The average elimination half life ($t_{\frac{1}{2}}\beta$) was 5.4 h and the $AUC_{0-\infty}$ was 3612 μg $ml^{-1}$ h. Pharmacokinetics were closely comparable to those reported in the Phase I studies (Kerr *et al.*, 1987). The mean AUC above the postulated activity threshold (Zaharko *et al.*, 1986) of 100 μg $ml^{-1}$ was 1865 μg $ml^{-1}$ h. A typical time course is illustrated in Figure 1.

FAA concentrations from the six melanoma tumour samples are shown in Table II. Analysis of divided tumours demonstrated excellent reproducibility. Average tumour concentrations achieved between the EOI and 65 min thereafter ranged between 122 and 183 μg FAA per gram. The sample taken 12 h after the EOI showed a mean tumour concentration of 20.1 μg $g^{-1}$. The tumour to plasma percentages for the samples from EOI to 65 min were closely grouped with a mean of 47.5 (± 7.9, SD)%. The value at 12 h was 25.7% in the single specimen analysed. The overall tumour to plasma percentage including the 12 h point was 43.9 (± 11.4, SD)%.

Concentrations were determined in plasma and tumour in mice bearing four different subcutaneous transplanted mouse tumours 30 m after 250 mg $kg^{-1}$ (750 mg $m^{-2}$) ip. Mean tumour levels ranged from 276–332 μg $g^{-1}$ and the tumour/plasma percentages were 48.6 ± 12.6 for EMT6, 62.7 ± 11.4 for RIF-1, 56.7 ± 5.9 for 16C and 58.5 ± 30.3 for KHT (± SD, $n$ = 5–8). Detailed pharmacokinetic parameters were determined (Figure 2a and Table III). Maximal tumour concentrations were achieved at 1 h (424.3 μg $g^{-1}$) with an

**Figure 1** A typical time course of plasma FAA concentration versus time following a 6 h infusion of 8.4 mg $m^{-2}$ in a patient with malignant melanoma.

**Table I** Plasma pharmacokinetic parameters in eight patients[a] receiving 8.6 g $m^{-2}$ FAA

| *Patient no.* | *Peak μg $ml^{-1}$* | *30 min μg $ml^{-1}$* | *$t_{\frac{1}{2}}\alpha$ min* | *$t_{\frac{1}{2}}\beta$ h* | *$AUC_{0-\infty}$ μg $ml^{-1}$ h* | *$AUC_{>100}$ μg $ml^{-1}$ h* | *Cl l $h^{-1}$* | *$Vd_{area}$ l* |
|---|---|---|---|---|---|---|---|---|
| 1 | 358.9 | 285.1 | 57.4 | 5.19 | 3005 | 1563 | 4.49 | 33.6 |
| 2 | 439.4 | 338.6 | 22.9 | 6.59 | 4992 | 2157 | 2.80 | 26.7 |
| 3 | 460.8 | 415.5 | 41.6 | 4.04 | 3976 | 2462 | 3.27 | 19.1 |
| 4 | 409.2 | 393.6 | 7.20 | 4.76 | 4476 | 2226 | 3.93 | 27.0 |
| 5 | 233.6 | 173.2 | 63.7 | 8.02 | 1998 | 5925 | 7.06 | 81.0 |
| 6 | 340.5 | 291.3 | 44.3 | 2.55 | 2795 | 1687 | 4.5 | 16.6 |
| 7 | 283.4 | 247.0 | 96.7 | 7.25 | 2383 | 1148 | 5.75 | 60.2 |
| 8 | 500.0 | 456.5 | 31.7 | 4.67 | 5268 | 3082 | 2.52 | 17.0 |
| Mean | 378.2 | 306.3 | 45.7 | 5.38 | 3612 | 1865 | 4.29 | 35.2 |
| SD | 91.07 | 84.16 | 27.5 | 1.80 | 1235 | 785 | 1.53 | 23.3 |

[a]Only two time points were available from patient 9.

**Table II** Tumour and plasma concentrations of FAA in human melanoma

| *Pt no.* | *Time after EOI* | *Tumour* ($\mu g^{-1}$) | *Tumour* (*mean*) | *plasma* ($\mu g\ ml^{-1}$) | *Tumour/plasma* (%) | *Tumour/plasma* (*mean*) |
|---|---|---|---|---|---|---|
| 8[a] | 0 | 196.8 | 183.4 | 390 | 50.5 | 47.1 |
| | | 157.3 | | | 40.4 | |
| | | 196.6 | | | 50.3 | |
| 7 | 0 | 114.1 | 131.8 | 283 | 40.2 | 46.6 |
| | | 123.0 | | | 43.4 | |
| | | 122.3 | | | 43.1 | |
| | | 165.4 | | | 58.4 | |
| | | 134.3 | | | 47.4 | |
| 9 | 10 min | 183.3 | 178.7 | 429 | 42.7 | 41.6 |
| | | 174.1 | | | 40.6 | |
| 6 | 22 min | 125.1 | 120.5 | 290 | 43.1 | 41.5 |
| | | 116.5 | | | 40.1 | |
| | | 119.9 | | | 41.3 | |
| 8[a] | 65 min | 169.0 | 176.3 | 290 | 58.3 | 60.8 |
| | | 183.6 | | | 63.4 | |
| 8[a] | 12 h | 14.5 | 20.1 | 78 | 18.5 | 25.7 |
| | | 20.1 | | | 25.7 | |
| | | 23.6 | | | 30.1 | |
| | | 22.4 | | | 28.7 | |

[a]Three samples taken from one patient after three different infusions.

elimination $t_{\frac{1}{2}}$ of 5.8 h. At 1–6 h the tumour/plasma % was constant with a mean of 73%. Thereafter, the tumour concentration fell more slowly than the plasma concentration, such that tumour exceeded plasma at these late times. Tumour/plasma percentages at 12 and 24 h after injection were 452% and 852% respectively. Similar results were obtained in a repeat experiment using 200 mg $kg^{-1}$ (600 mg $m^{-2}$) FAA (data not shown).

Parallel studies of KHT tumours after 200 mg $kg^{-1}$ (600 mg $m^{-2}$) ip FAA showed a rapid decline in the relative tumour blood flow. A nadir of 5% of control was observed at 6 h, followed by a slow recovery to 20% at 24 h (Figure 2B). No significant alteration occurred in a range of normal tissues (data not shown). Doses of 200 and 250 mg $kg^{-1}$ (600–750 mg $m^{-2}$) FAA ip resulted in regrowth delays of around 3–5 days in KHT sarcomas, compatible with the previously reported efficacy of FAA in murine tumours (Corbett *et al.*, 1986; Plowman *et al.*, 1986; Bibby *et al.*, 1988).

In contrast to plasma (Cummings *et al.*, 1988; Cummings & Smyth, 1989), no metabolites of FAA were detected in either human or mouse tumours.

**Table III** Pharmacokinetic parameters in mice with KHT tumours receiving 200 mg $kg^{-1}$ (600 mg $m^{-2}$) ip FAA

| | *Peak* $\mu g\ ml^{-1}$ | *Co* $\mu g\ ml^{-1}$ | $t_{\frac{1}{2}}$ *h* | $AUC_{0-\infty}$ $\mu g\ ml^{-1}\ h$ or $\mu g\ g^{-1}\ h$ | $AUC_{>100}$ $\mu g\ ml^{-1}\ h$ or $\mu g\ g^{-1}\ h$ |
|---|---|---|---|---|---|
| Plasma | 965.3 | 734.6 | 2.8 | 2985 | 1816 |
| Tumour | 431.0 | 348.3 | 5.8 | 2790 | 9834 |

### Discussion

One possible explanation for the marked discrepancy between the impressive effects of FAA against transplanted murine solid tumours (Corbett *et al.*, 1986; Plowman *et al.*, 1986; Bibby *et al.*, 1988) and the absence of activity in human studies (Kerr *et al.*, 1987; 1989; Kaye *et al.*, 1990) is a difference in tumour drug exposure between mice and humans. This study has shown a close parallel between the plasma exposures in the two species. Mean $AUC_{0-\infty}$ was 3612 $\mu g\ ml^{-1}$ h in the patients and 2985 $\mu g\ ml^{-1}$ h mice.

**Figure 2** FAA concentration versus time following ip injection of 250 mg $kg^{-1}$ (750 mg $m^{-2}$) in mice bearing KHT sarcomas. Open circles: plasma concentration (± 1SD) $\mu g\ ml^{-1}$; triangles: tumour concentrations (± 1SD) $\mu g\ g^{-1}$. A parallel experiment to show effects on tumour blood flow by rubidium extraction after ip injection of 200 mg $kg^{-1}$ (600 mg $m^{-2}$) FAA.

Similarly, above the postulated threshold of $100\,\mu g\,ml^{-1}$ (Zaharko *et al.*, 1986) the plasma drug exposures were highly comparable, being 1865 and $1816\,\mu g\,ml^{-1}\,h$ in humans and mice respectively. Tumour/plasma percentages were found to be marginally higher in the case of mouse tumours (48–63%) but of similar magnitude to that observed in humans (mean 47.5%) at early time points. The peak (30 min) tumour concentrations in murine tumours ($300\,\mu g\,g^{-1}$) were higher by a factor of 2 compared with the melanoma deposits over the first hour after EOI ($150\,\mu g\,g^{-1}$). After 6 h, there was a reproducible and marked elevation in the tumour/plasma percentages for the KHT tumour. This was not seen in the single melanoma biopsy obtained at 12 h, where the value was 25%. The elevation in the tumour/plasma percentages for the KHT tumour coincided with the abrupt fall in tumour blood flow, as noted previously in sensitive mouse solid tumours (Corbett *et al.*, 1986; Plowman *et al.*, 1986; Bibby *et al.*, 1988). Thus, it seems possible that the rise in tumour/plasma percentages was due to the trapping of FAA in KHT tumours as a result of the reduction of blood flow. Although it was only possible to obtain a single late time point in the human tumour study, the relatively low value there suggested that this trapping effect may not be seen in man.

It is particularly interesting to compare the exposures to FAA in the mouse and human tumours. The $AUC_{0-\infty}$ for the murine KHT tumour was $2790\,\mu g\,g^{-1}\,h$. This is similar to that of $1733\,\mu g\,g^{-1}\,h$ for the human melanomas estimated as the product of the average plasma $AUC_{0-\infty}$ and the mean tumour/plasma ratio of 0.48. The corresponding values above the putative activity threshold showed even closer agreement at $983\,\mu g\,g^{-1}\,h$ for the mouse KHT tumour and $895 \pm 377$(SD) $\mu g\,g^{-1}\,h$ for human melanomas. Thus the mouse and the predicted human FAA exposures are very similar. We have to emphasise however that we have only a single human melanoma value for time points beyond 1 h and further data for later times would be useful to confirm our prediction.

The mouse plasma and tumour exposures obtained in the present study are similar to those reported previously (Damia *et al.*, 1988; Chabot *et al.*, 1989). For example, Damia *et al.* (1988) observed a plasma $AUC_{0-\infty}$ ($\pm$SE) of $2021 \pm 166\,\mu g\,ml^{-1}\,h$ and a tissue/plasma % for the mouse PAN/03 tumour of 57% after $200\,mg\,kg^{-1}$ ($600\,mg\,m^{-2}$) FAA. The Mario Negri group also reported the only other published data for human tumour FAA levels (Damia *et al.*, 1990). They obtained biopsies of primary or metastatic tumour for six patients, in all cases 2 h after a 1 h infusion of $4.8\,g\,m^{-2}$ FAA in an EORTC ECTG study. Tumour/plasma percentages ranged from 25–80% and the mean of 45.8 ($\pm$24.5 SD)% was almost identical to the present study.

Taken together with previous experience, our studies appear to rule out inadequate tissue exposure as a cause of the lack of activity of FAA in human compared to mouse tumours. A species-specific mechanism, presumably involving the tumour vasculature or the immune system, may be responsible. Alternatively, the various effects of FAA seen in rodents may be a feature of transplantable tumours, rather than a species difference per se.

### References

BIBBY, M.C. (1991). Flavone acetic acid – an interesting novel therapeutic agent or just another disappointment? *Br. J. Cancer*, **63**, 3–5.

BIBBY, M.C., DOUBLE, J.A. & LOADMAN, P.M. (1988). Unique chemosensitivity of MAC 16 tumours to flavone acetic acid (LM975, NSC 347512). *Br. J. Cancer*, **58**, 341–344.

BIBBY, M.C., DOUBLE, J.A. LOADMAN, P.M. & DUKE, C.V. (1989). Reduction in tumour blood flow by flavone acetic acid: a possible component of therapy. *J. Natl Cancer Inst.*, **81**, 216–220.

CHABOT, G.G., BISSERY, M.-C., CORBETT, T.H., RUTKOWSKI, K. & BAKER, L.H. (1989). Pharmacodynamics and causes of dose-dependent pharmaco-kinetics of flavone acetic acid (LM-975; NSC-347512) in mice. *Cancer Chemother. Pharmacol.*, **12**, 15–22.

CORBETT, T.H., BISSERY, M.C. WOZNIAK, A & 5 others (1986). Activity of flavone acetic acid (NSC 347512) against solid tumours in mice. *Invest. New Drugs*, **4**, 207.

CUMMINGS, J., KERR, D.J., KAYE, S.B. & SMYTH, J.F. (1988). Optimisation of a reversed phase high performance liquid chromatographic method for the determination of flavone acetic acid and its major metabolites in plasma and urine. *J. Chromatog.*, **431**, 77–85.

CUMMINGS, J. & SMYTH, J.F. (1989). Flavone 8-acetic acid: our current understanding of its mechanism of action in solid tumours. *Cancer Chemother. Pharmacol.*, **24**, 269–272.

DAMIA, G., FRESCHI, A., SORIO, R. & 5 others (1990). Flavone acetic acid distribution in human malignant tumours. *Cancer Chemother. Pharmacol.*, **26**, 67.

DAMIA, G., ZANETTE, M.L., ROSSI, C., MANDELLI, R., FERRARI, A. & D'INCALCI, M. (1988). Dose dependent pharmacokinetics of flavone acetic acid in mice. *Cancer Chemother. Pharmacol.*, **22**, 47–50.

EVELOCH, J.L., BISSERY, M.-C., CHABOT, G.G. & 4 others (1988). Flavone acetic acid (NSC 347512) induced modulation of murine tumour physiology monitored by *in vivo* nuclear magnetic resonance spectroscopy. *Cancer Res.*, **48**, 4749.

FINLAY, G.J., SMITH, G.P., FRAY, L.M. & BAGULEY, B.C. (1988). Effect of flavone acetic acid on Lewis lung carcinoma: evidence for an indirect effect. *J. Natl Cancer Inst.*, **80**, 241–245.

GOUYETTE, A., KERR, D.J., KAYE, S.B. & 5 others (1988). Flavone acetic acid: a non linear pharmacokinetic model. *Cancer Chemother. Pharmacol.*, **22**, 114.

KAYE, S.B., CLAVEL, M., DODION, M., MONFARDINI, S. & 4 others (1990). Phase 2 trials with flavone acetic acid (NCS 347512, LM 975) in patients with advanced cancers of the breast, colon, head and neck and melanoma. *Investig. New Drugs*, **8**, S95.

KERR, D.J., KAYE, S.B., CASSIDY, J. & 7 others (1987). Phase I and pharmacokinetic study of flavone acetic acid. *Cancer Res.*, **47**, 6776.

KERR, D.J., MAUGHAN, T., NEWLANDS, E. & 4 others (1989). Phase II trials of flavone acetic acid in advanced malignant melanoma and colorectal carcinoma. *Br. J. Cancer*, **60**, 104.

MURRAY, J.C., SMITH, K.A. & THURSTON, G. (1989). Flavone acetic acid induces a coagulopathy in mice. *Br. J. Cancer*, **60**, 729–733.

PLOWMAN, J., NARAYANAN, V.L., DYKES, D. & 4 others (1986). Flavone acetic acid: a novel agent with preclinical antitumour activity against colon adenocarcinoma 38 in mice. *Cancer Treat Rep.*, **70**, 631.

SAPIRSTEIN, L.A. (1958). Regional Blood flow by fractional distribution of indicators. *Am. J. Physiol.*, **193**, 161–168.

URBA, W.J., LONGO, D.L., LOMBARDBO, F.A. & WEISS, R.B. (1988). Enhancement of natural killer activity in human peripheral blood by flavone acetic acid. *J. Natl Cancer Inst.*, **80**, 521–525.

WAGNER, G.J. (1975). *Fundamentals of Clinical Pharmacokinetics.* Hamilton: Drug Intelligence Publications.

WILTROUT, R.H., BOYD, M.R., BACK, T.C., SALUP, T.C., ARTHUR, J.A. & HORNUNG, R.L. (1988). Flavone-8-acetic acid augments natural killer cell activity and synergises with IL-2 for the treatment of murine renal cancer. *J. Immunol.*, **140**, 3261–3265.

WILTROUT, R.H. & HORNUNG, R.L. (1988). Natural products as antitumour agents: Direct versus indirect mechanisms of activity of flavonoids. *J. Natl Cancer Inst.*, **80**, 220–221.

WORKMAN, P. (1989). New drugs and novel agents. In *Current Opinions in Oncol.*, **1**, 213–221.

ZAHARKO, D.S., GREISHABER, C.K., PLOWMAN, J. & CRADOCK, J.C. (1986). Therapeutic and pharmacokinetic relationships of flavone acetic acid: an agent with activity against solid tumours. *Cancer Treat. Rep.*, **70**, 1415–1421.

ZWI, L.J., BAGULEY, B.C., GAVIN, J.B. & WILSON, W.R. (1989). Blood flow failure as a major determinant in the anti tumour action of flavone acetic acid. *J. Natl Cancer Inst.*, **81**, 1005–1013.